# Association between increased systemic immune-inflammation index and postoperative delirium in older intertrochanteric fracture patients

**DOI:** 10.1186/s13018-024-04699-8

**Published:** 2024-04-03

**Authors:** Xiaoxiao Yan, Jin Huang, Xiachan Chen, Mian Lin

**Affiliations:** 1 Department of Neurology, The Third Affiliated Hospital of Wenzhou Medical University, Wenzhou, People’s Republic of China; 2 Department of Orthopedics, The Third Affiliated Hospital of Wenzhou Medical University, Wenzhou, People’s Republic of China

**Keywords:** Systemic immune-inflammation index, Postoperative delirium, Inflammation, Older intertrochanteric fracture patients, Nomograms

## Abstract

**Background and purpose:**

The systemic immune-inflammation index (SII), a novel inflammation index derived from the counts of circulating platelets, neutrophils and lymphocytes, has been studied in the treatment of acute cancer and ischemic stroke (AIS). However, the clinical value of the SII in postoperative delirium patients has not been further investigated. The purpose of our research was to study the incidence and preoperative risk factors for postoperative delirium (POD) and verify whether the SII could serve as a potential marker for POD in older intertrochanteric fracture patients. Finally, we created a novel nomogram for predicting POD in older patients with intertrochanteric fractures.

**Methods:**

We enrolled elderly patients with intertrochanteric fractures who underwent proximal femoral nail antirotation (PFNA) between February 2021 and April 2023. Univariate and multivariate logistic analyses were subsequently performed to confirm the risk factors and construct a nomogram model.Calibration curve and clinical decision curve analysis (DCA) were used to assess the model’s fitting performance. The performance of the nomogram was evaluated for discrimination, calibration, and clinical utility.

**Results:**

A total of 293 patients were eligible for inclusion in the study, 25.6% (75/293) of whom had POD. The POD patients had higher SII values than the non-POD patients. The SII was strongly correlated with POD in older intertrochanteric fracture patients, and the optimal cutoff value was 752.6 × 10^9^. Multivariate analysis revealed that age, diabetes, total albumin, SII > 752.6 × 10^9^ and a CRP > 20.25 mg/L were independent risk factors for POD patients. By incorporating these 5 factors, the model achieved a concordance index of 0.745 (95% CI, 0.683–0.808) and had a well-fitted calibration curve and good clinical application value.

**Conclusion:**

The SII is a simple and valuable biomarker for POD, and the new nomogram model can be used to accurately predict the occurrence of POD. They can be utilized in clinical practice to identify those at high risk of POD in older intertrochanteric fracture patients.

## Introduction

Hip fracture is one of the most common and potentially devastating injuries in the elderly population. The incidence of this condition sharply increases in most areas of the world due to the aging of the population and the age-related increase in fractures [1, 2]. Specifically, femoral intertrochanteric fracture is the most common type of hip fracture. Surgical intervention, particularly proximal femoral nail antirotation (PFNA), is the preferred treatment for these fractures [3]. Previous studies have shown that intramedullary nails from various manufacturers provide comparable treatment outcomes [4]. In the context of elderly patients with intertrochanteric fractures, the primary challenge lies not in the surgical procedure itself but rather in perioperative management aimed at mitigating the risk of complications, such as pulmonary embolism, pneumonia, and delirium [1]. Delirium is characterized by disturbed attention, consciousness and other cognitive abilities. Postoperative delirium (POD) is one of the most common complications following surgery. POD has a negative effect on postoperative recovery, increases the nursing burden of caregivers, extends the length of hospital stay, increases hospitalization costs and hospitalization mortality, and may even lead to long-term cognitive impairment [5–8]. POD usually occurs within the first 72 h after surgery and lasts for several days [9]. Studies have shown that the incidence of POD is between 15.7 and 48% in elderly patients after hip fractures [5, 10, 11]. There is much evidence suggesting that POD is associated with neuroinflammatory processes [12]. Several inflammatory markers in serum or cerebrospinal fluid have been proven to be closely associated with POD [13]. The systemic immune-inflammation index (SII), a relatively novel inflammatory index that combines peripheral lymphocyte, neutrophil, and platelet counts, can be obtained without any additional financial burden. A growing body of studies has shown the potential prognostic value of POD for head and neck free-flap reconstruction, abdominal surgery, esophagectomy, carotid endarterectomy and cardiac surgery [14–18]. However, few studies have shown that the SII can be used to predict postoperative delirium (POD) in older hip fracture patients, particularly those with intertrochanteric fractures. It is crucial to establish a specialized model to identify high risk patients as early as possible and further reduce the incidence of POD with intertrochanteric fracture for PFNA. In our single-center retrospective cohort study, we investigated the value of the SII in predicting POD in older intertrochanteric fracture patients. Ultimately, we aimed to create a nomogram prediction model to assist clinicians in accurately identifying POD in elderly patients with intertrochanteric fractures who under PFNA.

## Methods

### Study population

This study was a retrospective cohort study. All the data were obtained from patients at the Third Affiliated Hospital of Wenzhou Medical University from February 2021 to April 2023.

Patients were included in the study only if they met all the following criteria: 1) aged ≥ 60 years; 2) had clinically and radiographically confirmed intertrochanteric fractures and agreed to participate in PFNA surgery; 3) American Society of Anesthesiologists’ physical status I–III; 4) had spinal anesthesia.

**Fig. 1:**
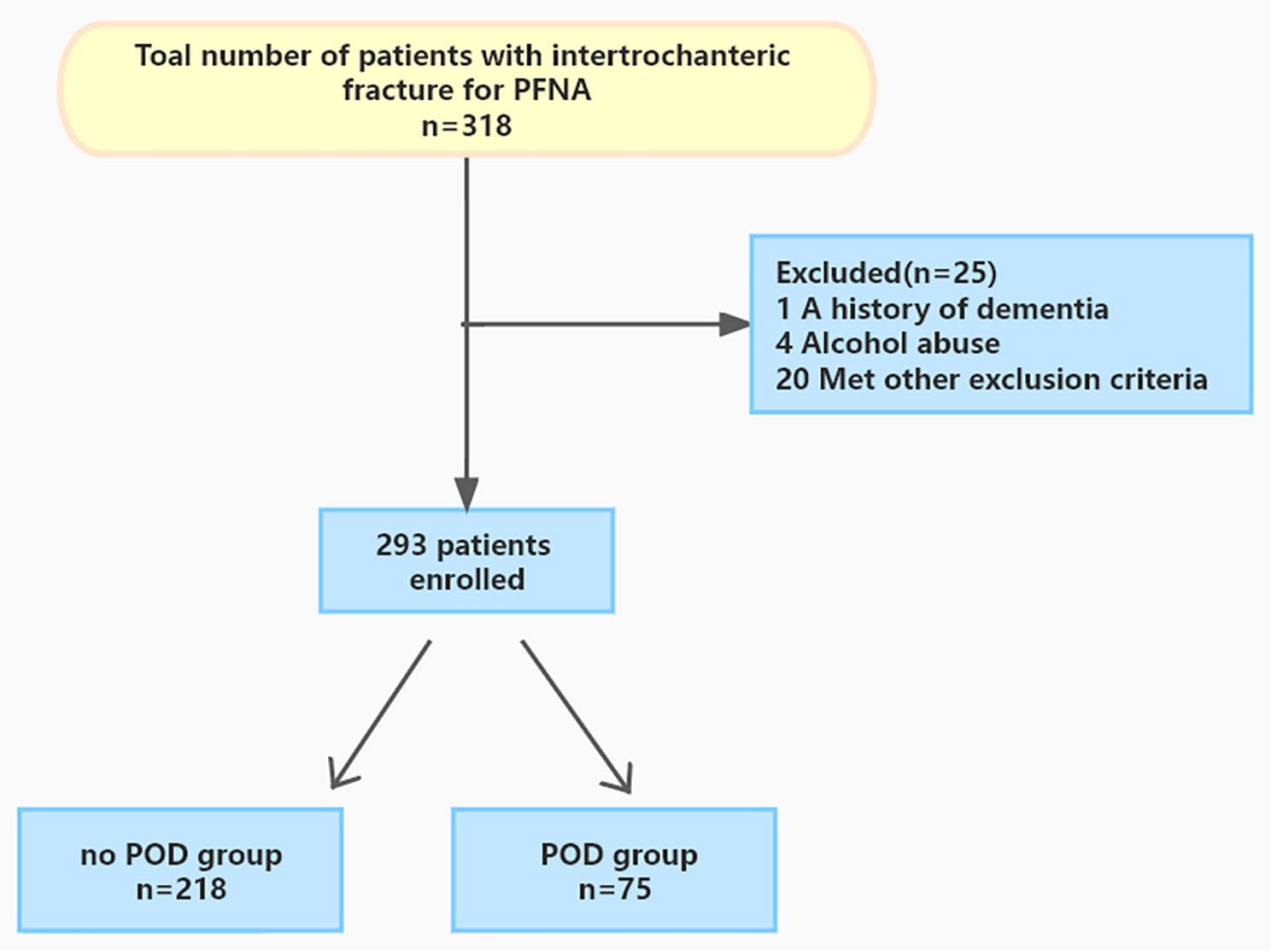
Flow chart for patient selection

The exclusion criteria were as follows: 1) an infection at admission; 2) postoperative infections; 3) multiple injuries or multiple fractures; 4) a history of dementia or mental illness; 5) patients who had undergone other surgeries within 6 months; 6) cardiac disease (including acute myocardial infarction, congestive heart failure, a history of tachyarrhythmia/bradyarrhythmia or atrial fibrillation), pulmonary disease and impaired renal function (estimated glomerular filtration rate < 60 mL/min per 1.73 m^2^); 7) using antipsychotic medications; 8) alcohol or drug abuse; 9) incapable of appropriate communication; 10) preoperative delirium, stroke after surgery and in-hospital mortality. Overall, 293 patients were included in our study. Figure 1 presents the selection of patients in a flow chart.

This study was approved by the Ethics Committee of the Third Affiliated Hospital of Wenzhou Medical University (LZM2023005) and was carried out in accordance with the Declaration of Helsinki. Because this study was retrospectively designed, the ethics committee granted a waiver of the requirement for informed consent regarding the use of existing data in accordance with the national legislation and the institutional requirements. For the purpose of privacy protection in this study, personal identification information of the enrolled participants was anonymized and replaced with a coding system.

### Data collection

Patient data such as age, sex, smoking status, body mass index, education degree level, hypertension, diabetes, postoperative analgesic pump use and American Society of Anesthesiologists (ASA) physical score were obtained at the preoperative visit. Surgical information, including surgical duration and blood loss was recorded.

Laboratory data, including hematocrit, neutrophil count, lymphocyte count, platelet count, total albumin and C-reactive protein (CRP) level, which were most recently measured prior to surgery and measured within 24 h after admission, were collected.SII was defined as platelet neutrophil/lymphocyte ratio.

Symptoms and signs of infection were estimated, and white blood cell count, procalcitonin (PCT) level, urine screening, chest CT, and body temperature (at least 3 times a day) were monitored within 7 days after admission.Patients with infections such as urinary tract infection, pulmonary infection, or infections in other locations were excluded.

### Definitions of outcomes

The primary outcome was the incidence of POD within seven days postoperatively. It was captured through descriptive words documented in the medical records and confirmed by the neurologist. The inclusion criteria were as follows: (1) postoperative medical records containing “mental status change,” “confusion,” “disorientation,” “agitation,” “delirium,” “inappropriate behavior,” “inattention,” “hallucinations,” and “combative behavior“ [19]; and (2) postoperative drug regimens including “quetiapine,” “olanzapine,” “haloperidol,” “haloperidol,” and “risperidone.” Neurologists rechecked all the delirium patients’ medical records for diagnosing POD using the Diagnostic and Statistical Manual of Mental Disorders, fourth edition (DSM-IV) criteria [14].

### Statistical analysis

Patients were classified into non-POD and POD groups. We used the mean ± standard deviation (SD) to describe continuous variables with a normal distribution and the median to describe continuous variables with a nonnormal distribution. To identify differences between non-POD and POD groups, the Pearson χ2 test was used for categorical variables. Student’s t test was used to compare normally distributed variables. Mann‒Whitney U tests were used to compare non-normally distributed variables. These variables were included in multivariate logistic regression analysis to determine whether the SII was an independent predictor of POD. Variables associated with POD in the univariate analyses with a *P value < 0.05* were included in the multivariate analysis. The results are expressed as the adjusted odds ratio (aOR) with the corresponding 95% confidence interval (CI). A nomogram model was constructed based on multivariate analysis through the “R” package. Receiver operating characteristic (ROC) curve analysis, calibration curve analysis, and clinical decision curve analysis (DCA) were conducted using the “proc,” “resource selection,” and “rmda” packages. A *P value < 0.05* indicated that the difference was statistically significant. The data were analyzed using IBM SPSS Statistics (26.0) and R software (4.1.3).

## Results

### Characteristics of the study subjects

Our study enrolled 293 intertrochanteric fracture patients; the mean age was 80.34 ± 9.61 years (60–98 years), and 49.5% (145) of the patients were women. In the study population, 172 patients had a history of hypertension, 105 had a history of diabetes, and 74 patients were smokers. The mean surgery duration was 55.03 ± 17.33 min, and blood loss was 110.17 ± 19.80 ml. The baseline characteristics are displayed in Table 1.

**Table 1:** Comparison of baseline characteristics between the non-POD and POD groups

	Non-POD group (218)	POD group (75)	*P*
Age, y (Mean SD)	79.68 ± 9.88	82.24 ± 8.57	***< 0.05***
Female, n(%)	105(48.2)	40(53.3)	0.440
Male, n(%)	113(51.8)	35(46.7)	0.440
BMI ≥ 24 kg/m, n(%)	21(9.63)	5(6.67)	0.436
Current smoking, n(%)	54(24.8)	20(26.7)	0.744
Degree of education			0.346
Illiteracy	157(72.0)	49(65.3)	
Primary school	46(21.1)	22(29.3)	
Other	15(6.9)	4(5.4)	
Hypertension, n(%)	127(58.3)	45(60.0)	0.791
Diabetes, n(%)	70(32.1)	35(46.7)	***< 0.05***
ASA grading			0.820
II	215(98.7)	73(97.3)	
III	3(1.3)	2(2.7)	
Surgery duration (mean SD), min	54.62 ± 17.21	56.24 ± 17.75	0.486
Blood loss (mean SD), ml	109.76 ± 20.60	111.36 ± 17.35	0.547
Postoperative analgesic pump use	142(65.1%)	50(66.6%)	0.810
Neutrophil count (×10^9^/L), (Mean SD)	4.63 ± 2.38	5.16 ± 1.99	0.082
Lymphocyte count (× 10^9^/L), (Mean SD)	1.41 ± 0.50	1.27 ± 0.39	***< 0.05***
Platelet count (× 10^9^/L), (Mean SD)	159.39 ± 58.82	175.50 ± 65.08	0.069
SII (10^9^/L)	628.86 ± 616.81	788.39 ± 528.49	***< 0.05***
CRP (mg/L), (Mean SD)	21.29 ± 12.23	24.58 ± 11.96	***< 0.05***
Hemoglobin(g/dl)	101.56 ± 16.61	101.17 ± 17.76	0.868
Total albumin(g/L)	35.29 ± 3.65	34.04 ± 4.51	***< 0.05***

*P* values were calculated by the Chi-square test, Fisher’s exact test, the Mann‒Whitney U test or the t test

POD, postoperative delirium; BMI, body mass index; ASA, American Society of Anesthesiologists; CRP, C-reactive protein; SII = systemic immune-inflammation index; bold text indicates *P* values less than 0.05

A total of 75 (25.6%) patients experienced POD, and patients with POD were significantly older (82.24 ± 8.57 vs. 79.68 ± 9.88, *P* < 0.05), had a greater percentage of diabetes (46.7% vs. 32.1%, *P* < 0.05), had a greater SII [788.39 ± 528.49 vs. 628.86 ± 616.81, *P* < 0.05], had a greater CRP level (24.58 ± 11.96 vs. 21.29 ± 12.23, *P* < 0.05), had a lower lymphocyte count (1.27 ± 0.39 vs. 1.41 ± 0.50, *P* < 0.05) and had a lower total albumin (34.04 ± 4.51 vs. 35.29 ± 3.65, *P* < 0.05).

### Evaluation of the prognostic value of the SII and CRP for POD

**Fig. 2:**
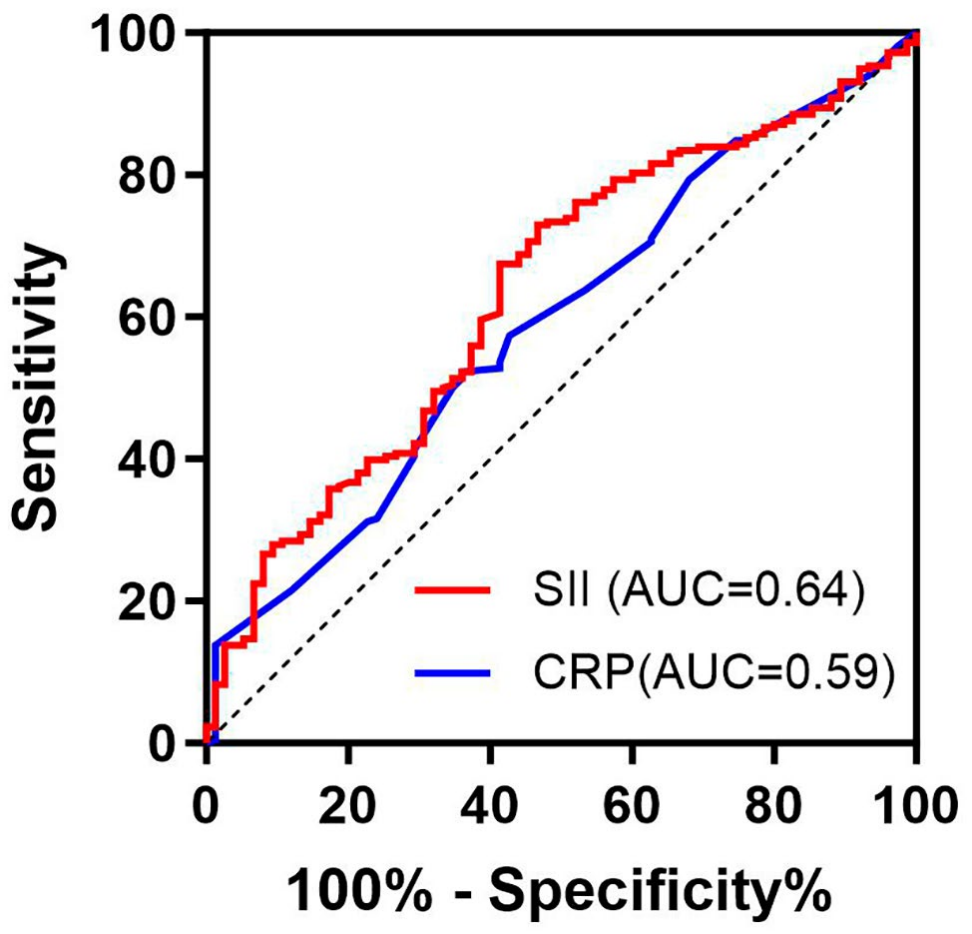
Evaluation of the prognostic value of the SII and CRP for POD

ROC curve analysis revealed that the SII was predictive of POD, with an AUC of 0.64 (95% CI 0.56 to 0.70), and that of CRP was 0.59 (95% CI 0.52 to 0.66) (Fig. 2). The SII cutoff value was 752.6 × 10^9^, the sensitivity was 72.3%, and the specificity was 53.3%; however, the CRP cutoff value was 20.25 mg/L, the sensitivity was 64.00%, and the specificity was 52.5%. The proportion of patients with an SII ≥ 752.6 × 10^9^ was significantly greater than that of patients without delirium (39 [52.0%] vs. 60 [27.5%], *P* < 0.001).

### Multivariate analysis for risk factors

Unadjusted logistic regression analysis revealed that age, diabetes, lymphocyte count, total albumin, the SII and the CRP level were correlated with POD. The 6 variables with *P* values less than 0.05 were included in the multifactorial analysis, and the results are shown in Table 2.Age (OR = 1.034, 95% CI: 1.002–1.067, *P* = 0.035), diabetes(OR = 2.450, 95% CI: 1.330–4.511, *P* = 0.047), total albumin(OR = 0.903, 95% CI: 0.837–0.974, *P* = 0.004), an SII > 752.6 × 10^9^ (OR = 0.311, 95% CI: 0.158–0.614, *P* = 0.001) and a CRP concentration > 20.25 mg/L (OR = 0.463, 95% CI: 0.256–0.837, *P* = 0.011) were found to be independent predictors of POD.

**Table 2:** Multivariate analysis of risk factors

	OR (95% CI)	*P*
Age	1.034(1.002–1.067)	*< 0.05*
Diabetes	2.450(1.330–4.511)	*< 0.05*
Total albumin	0.903(0.837–0.974)	*< 0.05*
SII > 752.6 × 10^9^	0.311(0.158–0.614)	*< 0.05*
CRP > 20.25	0.463(0.256–0.837)	*< 0.05*

### Development and verification of nomogram model

**Fig. 3:**
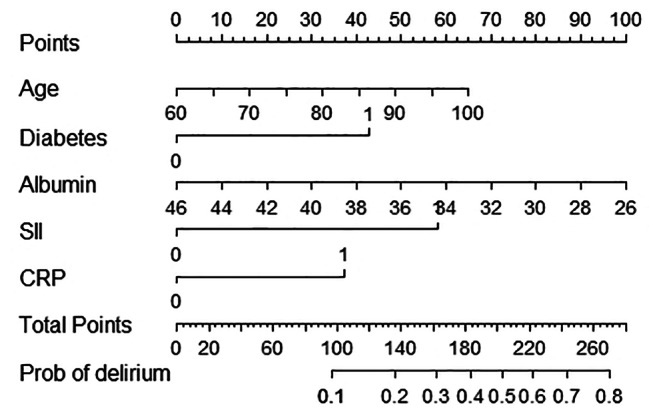
Nomogram for predicting POD risk based on the systemic immune-inflammatory index (SII)

**Fig. 4:**
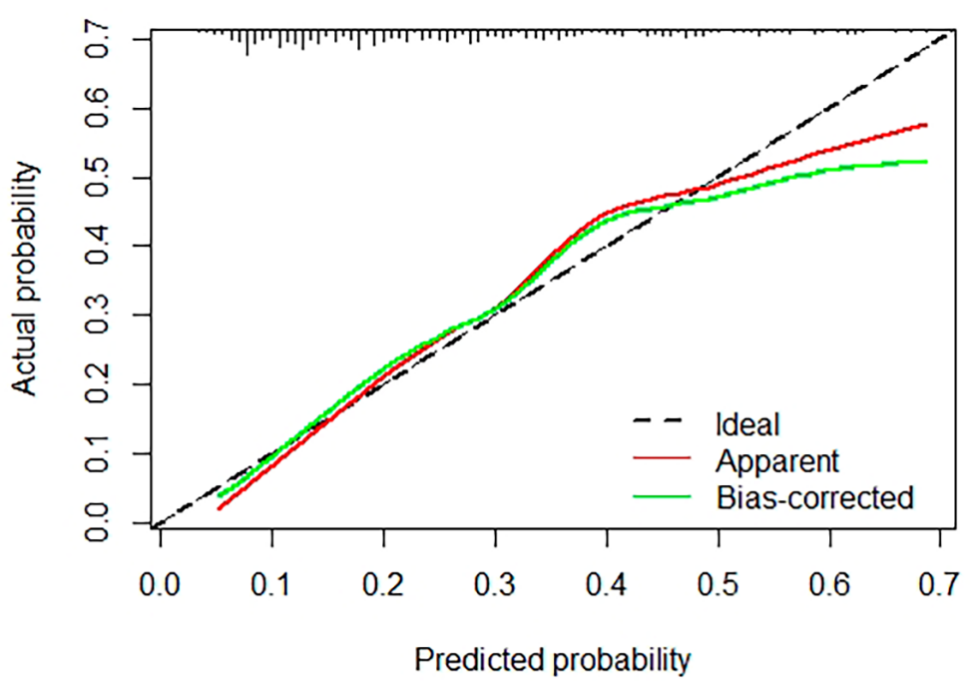
Calibration plot of the nomogram for POD (bootstrap 1000 repetitions)

**Fig. 5:**
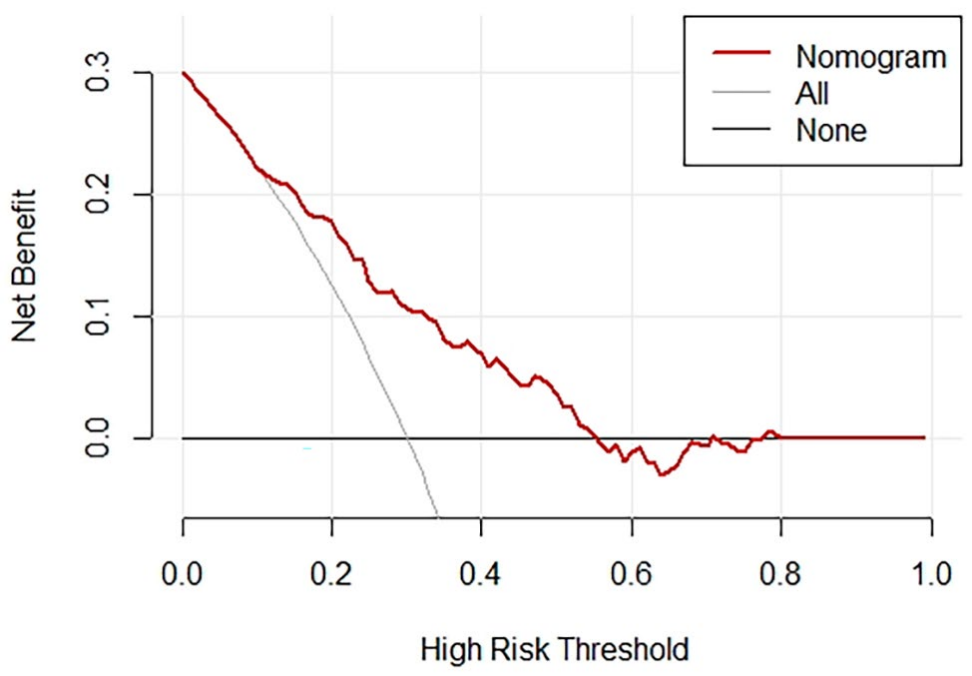
Decision curve analysis (DCA) of the nomogram for POD

**Fig. 6:**
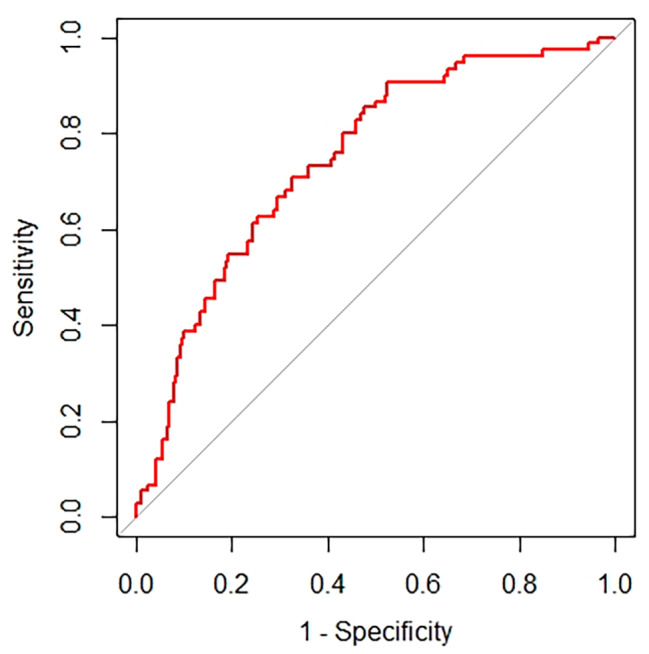
The receiver operating characteristic (ROC) curve of the nomogram

Using multivariate analysis, a nomogram prediction model for POD was built and is displayed in Fig. 3. The corresponding predicted risk of POD was evaluated with a total score of 5 items. A reasonable calibration was confirmed by the Hosmer‒Lemeshow goodness-of-fit test (*p* = 0.559), and the results are displayed in Fig. 4. The net benefit associated with the use of the nomogram model is presented as a decision curve analysis (DCA) curve in Fig. 5. The bootstrap method (*n* = 1000) was used for internal validation of the nomogram, and the area under the ROC curve (AUC) was 0.745 (95% CI, 0.683–0.808) as shown in Fig. 6.

## Discussion

Chronic stress induced by surgery, anesthesia, or trauma often nonspecifically activates the immune system. This activation is characterized by an increase in neutrophil count and a decrease in lymphocyte count, along with a decrease in platelet count in peripheral circulation [20–22]. This activation may involve hypercortisolism, disruption of the blood‒brain barrier, activation of microglia, and release of cerebral cytokines, which may contribute to the pathophysiology of delirium [22, 23]. Several recent studies have shown that white-cell-derived inflammatory biomarkers, including the neutrophil-to-lymphocyte ratio (NLR) and the systemic immune-inflammation index (SII), are indeed correlated with postoperative delirium (POD). A prospective multicenter study conducted in three medical centers enrolled 182 patients with POD and revealed a correlation between the NLR and POD in elderly patients who underwent THA for hip fracture. Compared to patients without POD, those with an NLR ≥ 3.50, older age, diabetes, and a higher neutrophil count were associated with an increased risk of POD [24]. Yuxiang Song et al. observed a total of 29,608 patients who did not undergo neurosurgery or cardiac surgery, among whom the SII was an independent predictor. They found that the SII was significantly greater in patients with POD, and the SII was found to be a useful prognostic predictor of POD in patients of different ages, sexes, and ASA classifications [24].To the best of our knowledge, intertrochanteric femoral fracture is the most commonly observed fracture in the elderly population, accounting for 63% of all hip fractures [25]. However, to our knowledge, the SII has not been applied in elderly patients with intertrochanteric fractures, who have a high incidence of POD. Therefore, this retrospective study is the first to investigate the relationship between POD and the SII. In our study, 25.6% (75/293) of older intertrochanteric fracture patients developed POD, similar to previous studies [26–28]. The ROC curve indicated an AUC of 0.64 for the preoperative SII, with an optimal cutoff value of 752.6 × 10^9^. This suggests that patients with an SII > 752.6 × 10^9^ are at a greater risk of developing POD. Logistic regression analysis revealed that a high SII, advanced age, diabetes, total albumin, and CRP concentration > 20.25 mg/L were independent risk factors for POD in older patients with intertrochanteric fractures.

The results of this study indicate that age is an independent risk factor for POD, consistent with previous studies [24, 28–30]. Wang et al. Conducted multiple logistic regression analysis on 582 patients who underwent joint surgery and found that age was a significant risk factor for postoperative delirium. The risk was significantly greater in patients older than 70 years than in those younger than 70 years [30]. Aging leads to a decline in organ compensatory function and physical fitness, a decrease in the brain’s regulatory ability, increased susceptibility to stressors, and abnormal excitation conduction ability, all of which contribute to the development of delirium. Additionally, aging results in a decrease in brain volume, loss of synapses in nerve cells, elevated cortisol levels, reduced oxyacetylene levels, sleep disturbances, and a gradual decline in memory and attention, all of which can cause POD [31].

Diabetes mellitus has been recognized as a risk factor for postoperative delirium (POD) in previous studies. In our study, patients with diabetes mellitus had a 2.45-fold greater risk of POD than patients without diabetes mellitus. Recent research has revealed a close association between diabetes and cognitive dysfunction, including dementia and Alzheimer’s disease. This association may be driven by factors such as insulin resistance, altered glucose metabolism, vascular changes, and the metabolism of β-amyloid and tau [32–34].In our study, the relevant *p* value (*P* = 0.047*)* was unfortunately close to 0.05, which may be attributed to the small sample size.

In our study, we also found that elderly patients with intertrochanteric fractures and hypo-protein status had a relatively high incidence of POD. Quinlan GJ et al. reported that albumin functions as an antioxidant, scavenges metabolites, protects the microcirculation, and promotes drug binding and transport [31], supporting our results. Elderly patients have reduced albumin levels, which reflects their poor nutritional status. In addition, the production process leads to an increase in oxygen free radicals that cannot be efficiently cleared, resulting in damage to brain cells through the blood‒brain barrier. A decrease in albumin levels can weaken the binding ability of drugs, such as those that cause central nervous system side effects. This can increase the concentration of free drugs in the body and potentially lead to mental disorders. Therefore, it is particularly important for doctors to identify the causes of hypoproteinaemia and actively correct the state of hypoalbuminemia to prevent the occurrence of POD.

There is a growing body of evidence supporting a link between CRP levels and postoperative delirium in various settings [35–39]. Chantal J et al. suggested that CRP plays a role in the underlying (inflammatory-vascular) pathological pathway of postoperative delirium [39]. Sarinnapha M et al. conducted a study suggesting that preoperative CRP could serve as an important risk marker for delirium incidence and that CRP measured preoperatively could aid in predicting and monitoring the severity of POD [40]. These findings are consistent with our own research. The SII also represents the systemic immune-inflammation status. In this study, the ROC curve and logistic regression analyses demonstrated that the SII had greater sensitivity and specificity than the CRP. Specifically, the SII was 0.64, while the AUC of CRP was 0.59, proving that the SII showed good predictive power for POD.

The SII was strongly correlated with POD. Unlike in previous studies on risk factors, independent risk factor data were obtained only by combining the Chi-square test, t test and other methods as well as univariate logistic regression analysis. The independent risk factors identified in this study were more accurately analyzed by univariate and multivariate logistic regression. Additionally, we developed a nomogram model and internally validated its excellent diagnostic efficacy. Different scores were obtained for each variable, after which the total scores of the 5 items were assessed for the corresponding POD-predicted risk. The calibration plot and the Hosmer‒Lemeshow goodness-of-fit test indicated that the model’s calibration was reasonable (*p* = 0.559). The DCA curve in Fig. 5 illustrates that the nomogram model has excellent clinical application. The internal verification of the nomogram was performed through the bootstrap method with 1000 repetitions of sampling, and the AUC was 0. 745, indicating that the model had good prediction performance. The new nomogram score can be derived and used by clinicians to predict the occurrence of POD in older intertrochanteric fracture patients. This tool could be helpful for clinicians by enabling early evaluation of patient outcomes, thereby aiding in the selection of better treatment plans.

However, our study has several limitations as follows. First, our study is a single-center study with a relatively small sample size, which may cause selection bias and inaccuracy to some degree. Patients with POD were identified based on medical and nursing records but not assessment tools. Nurses may have missed delirium because of a lack of knowledge of POD assessment. Second, we investigated only the SII values at admission, while the dynamic variability over time should also be assessed and studied. Third, we tried to reduce the impact of confounding factors on outcomes, but confounding factors could still not be completely ruled out in the multiple logistic regression analysis. Finally, there was a shortage of sufficient external validation targeting the new predictive model. Considering the above limitations, some prospective clinical trials with sufficient samples need to be designed in the future to evaluate the diagnostic and prognostic value of the SII for POD in older intertrochanteric fracture patients.

## Conclusion

The SII is a straightforward and valuable predictor of POD in older intertrochanteric fracture patients. The new nomogram model, which consisted of 5 risk factors, provided an intuitive and accurate prediction of POD. It could be used in clinical practice to identify intertrochanteric fracture patients with PFNA who are at high risk of postoperative delirium.

## Data Availability

No datasets were generated or analysed during the current study.
